# Creep-Fatigue Crack Initiation Simulation of a Modified 12% Cr Steel Based on Grain Boundary Cavitation and Plastic Slip Accumulation

**DOI:** 10.3390/ma14216565

**Published:** 2021-11-01

**Authors:** Xin Jin, Run-Zi Wang, Yang Shu, Jia-Wen Fei, Jian-Feng Wen, Shan-Tung Tu

**Affiliations:** School of Mechanical and Power Engineering, East China University of Science and Technology, Shanghai 200237, China; xinjin680912@126.com (X.J.); rzwang@ecust.edu.cn (R.-Z.W.); shu330814@sina.com (Y.S.); FFEIJW@163.com (J.-W.F.); sttu@ecust.edu.cn (S.-T.T.)

**Keywords:** creep, fatigue, crack initiation, crystal plasticity, cavity growth

## Abstract

High-temperature components in power plants may fail due to creep and fatigue. Creep damage is usually accompanied by the nucleation, growth, and coalescence of grain boundary cavities, while fatigue damage is caused by excessive accumulated plastic deformation due to the local stress concentration. This paper proposes a multiscale numerical framework combining the crystal plastic frame with the meso-damage mechanisms. Not only can it better describe the deformation mechanism dominated by creep from a microscopic viewpoint, but also reflects the local damage of materials caused by irreversible microstructure changes in the process of creep-fatigue deformation to some extent. In this paper, the creep-fatigue crack initiation analysis of a modified 12%Cr steel (X12CrMoWvNBN10-1-1) is carried out for a given notch specimen. It is found that creep cracks usually initiate at the triple grain boundary junctions or at the grain boundaries approximately perpendicular to the loading direction, while fatigue cracks always initiate from the notch surface where stress is concentrated. In addition to this, the crack initiation life can be quantitatively described, which is affected by the average grain size, initial notch size, stress range and holding time.

## 1. Introduction

The increasing industrial demand of efficiency puts forward higher requirements for the service conditions of process equipment [1]. Many components used in petrochemical, power and energy, aerospace and other industries need to be in service for a long time under harsh environment and complex loading conditions [2]. Damage and failures, however, occurs frequently in these components [3,4,5,6,7]. X12CrMoWvNBN 10-1-1 steel, for example, has excellent high-temperature properties and is widely used in ultra-supercritical units, but it is vulnerable to creep and creep-fatigue damage [8,9,10]. It is thus necessary to understand its creep and/or-fatigue damage behavior.

In recent years, the creep-fatigue damage mechanisms [11,12,13] and modeling approaches [14,15,16,17] have been extensively investigated. For instance, Danilov et al. [18] studied the evolution of the plastic strain macro-localization pattern in low-temperature creep of commercial purity aluminum, and they found that the velocity of plastic strain localization waves is governed by thermally activated dislocation movement. Yasniy et al. [19] analyzed the effects of frequency and loading waveform on FCG rate in bimetal of continuous caster rolls with fracture mechanics, where a unilateral accumulation of plastic deformation and a crack tip blunting leads to a reduction of the crack propagation rates. Regarding the crystal plasticity theory, Busso et al. [20] developed a gradient- and rate-dependent crystallographic formulation considering effective obstacle spacings. Sauzay and Jourdan [21] investigated the mechanism of high cycle fatigue crack initiation in polycrystalline materials, and they found that grain orientation plays a critical role in the activation of the slip system and the subsequent crack initiation. Simonovski et al. [22] looked into the effects of crystal plasticity on grain stiffness and concluded that the highest Schmid factors lead to the maximal crack tip opening displacement. McDowell and Dunne [23] explored the sensitivity of fatigue crack initiation to microstructure using a crystal plasticity finite element model. Further, Li et al. [24] developed a model that represents the geometry of grains, sub-grains and precipitates and well predicted the lifetime of fatigue crack initiation using an accumulated slip parameter considering the stress triaxiality. The results show that coarsening of precipitates has a detrimental effect on fatigue at elevated temperature. Tang et al. [25] developed a crystal plasticity model to simulate geometrically necessary dislocations (GNDs) in a titanium alloy under high-cycle fatigue loading. The simulation results were consistent with the density distribution of GNDs after high-cycle fatigue loading characterized by electron backscatter diffraction (EBSD). Efthymiadis et al. [26] discussed the advantages and disadvantages of fatigue crack initiation criteria proposed by others and developed an approach combining experimental and modelling. Liu and Pons [27] proposed a microstructural conceptual framework, wherein fatigue and creep are treated separately due to different damage principles. This conceptual framework is consistent with the various creep and fatigue microstructural observations in the literature.

However, few studies have implemented both creep and fatigue meso-damage mechanisms into crystal plasticity modeling, nor have they distinguished contributions of the two physical mechanisms to crack initiation at a microstructural level. Normally, the creep-fatigue damage was only estimated by a simple addition of each damage variable at macroscale, even if the addition may be unreasonable. Based on the crystal plasticity theory, this paper introduces a creep meso-damage mechanism with nucleation, growth and coalescence of grain boundary cavities under the framework of the rate-dependent cyclic constitutive model and combines the equivalent cumulative plastic strain to predict fatigue crack initiation. Simultaneously, the location and influencing factors of creep and fatigue crack initiations are fully explored through finite element simulations of notched specimens.

## 2. Model

Under the framework of the rate-dependent constitutive model, the creep meso-damage model considering grain boundary voids nucleation, growth and coalescence is introduced. Meanwhile, the equivalent accumulated plastic strain is used as the fatigue indicator to predict the initiation of fatigue cracks. The finite element implementation of the model in ABAQUS is realized by developing a UMAT subroutine that calculates damages in grains and at grain boundaries.

### 2.1. Crystal Plasticity Model

This section briefly describes the finite strain framework of an anisotropic constitutive model. The model can simulate the deformation mechanism of the material microstructure under creep condition from a microscopic point of view.

For better describing the constitutive relation of single crystal deformation, the total deformation gradient F is often assumed to be a multiplicative decomposition of elastic and plastic parts:

(1) $$F=F^{e}F^{p}$$

where $F^{p}$ is the plastic part of the total deformation from the reference configuration to the intermediate configuration caused only by dislocation slip, and $F^{e}$ is the elastic deformation imposed on the intermediate configuration, including the tensile deformation and rotational deformation of the crystal lattice. The velocity gradient **L** in the current configuration can be given by:

(2) $$L=\dot{F}F^{-1}={\dot{F}}^{e}F^{e-1}+F^{e}{\dot{F}}^{p}F^{p-1}F^{e-1}=L^{e}+L^{p}$$

where $L^{e}$ and $L^{p}$ respectively represent the elastic and plastic part of velocity gradient. It can be considered that the inelastic deformation of a metal single crystal is similar to the simple shear motion on a slip system, so the plastic velocity gradient depends linearly on crystallographic slip rate as follows:

(3) $$L^{p}=\sum_{\alpha =1}^{n}{\dot{\gamma }}^{\alpha }s^{\alpha }⊗m^{\alpha }$$

where *n* refers to the total number of slip systems, $s^{\alpha }$,$m^{\alpha }$ and ${\dot{\gamma }}^{\alpha }$ are the slip direction, slip plane normal and slip strain rate of slip system *α*, respectively. The resolved shear stress $\tau^{\alpha }$ can be expressed by the Cauchy stress tensor as:

(4) $$\tau^{\alpha }=\mathrm{symm}\left[s^{\alpha }⊗m^{\alpha }\right]:\sigma$$

where ⊗ indicates a tensor product, symm represents the symmetric part of the tensor, and the slip direction $s^{\alpha }$ and the normal direction of the slip plane $m^{\alpha }$ are defined in the current configuration. The transformation relationship between the slip direction and the normal direction of the slip plane in the reference configuration and the current configuration can be expressed as:

(5) $$s^{\alpha }=F^{e}s_{0}{}^{\alpha },m^{\alpha }=m_{0}{}^{\alpha }F^{e-1}$$

where $s_{0}{}^{\alpha }$ and $m_{0}{}^{\alpha }$ represent the slip direction and the normal of the slip plane in the slip system α in the reference configuration, respectively. The flow rule is assumed to follow a form of a viscoplastic power-law expression:

(6) $${\dot{\gamma }}^{\alpha }={\dot{\gamma }}_{0}{\left\{\left|\frac{\tau^{\alpha }-\chi^{\alpha }}{g^{\alpha }}\right|\right\}}^{m}\mathrm{sgn}\left(\tau^{\alpha }-\chi^{\alpha }\right)$$

where $g^{\alpha }$ is the slip resistance function of isotropic hardening, $\chi^{\alpha }$ is the back stress of kinematic hardening, *m* is the rate-sensitive exponent, ${\dot{\gamma }}_{0}$ is the reference shear strain rate, and sgn is the symbolic function.

The hardening law of $g^{\alpha }$ and $\chi^{\alpha }$ can be obtained by using the Armstrong-Frederick hardening rule [28,29,30]:

(7) $${\dot{g}}^{\alpha }=\sum_{\beta }h_{\alpha \beta }{\dot{\gamma }}^{\beta }$$

(8) $${\dot{\chi }}^{\alpha }=c{\dot{\gamma }}^{\alpha }-d\chi^{\alpha }\left|{\dot{\gamma }}^{\alpha }\right|$$

where c and d are the direct hardening coefficient and dynamic recovery coefficient, and $h_{\alpha \beta }$ is the hardening matrix. The diagonal term $h_{\alpha \alpha }$ defines self-hardening modulus and off-diagonal term $h_{\alpha \beta }\left(\alpha \neq \beta \right)$ refers to latent hardening modulus, which can be further written as:

(9) $$h_{\alpha \beta }=\{\begin{array}{cc} h(\gamma )=h_{0}{\mathrm{sech}}^{2} & \left|\frac{h_{0}\gamma }{\tau_{s}-\tau_{0}}\right|,\;\alpha =\beta \\ qh(\gamma ), & \alpha \neq \beta \end{array}$$

where q and $h_{0}$ are the latent hardening parameter and the initial hardening modulus, and $\tau_{0}$ is the critical value of the initial resolved shear stress, also called the initial slip system strength when γ=0 and $g^{\alpha }=\tau_{0}$.$\tau_{s}$ is the critical value of the saturated resolved shear stress, which is the critical value of all active slip systems. The symbol sech denotes the hyperbolic secant function. The total accumulated slip γ is expressed as follows:

(10) $$\gamma =\sum_{\alpha =1}^{n}\int_{0}^{t}\left|{\dot{\gamma }}^{\alpha }\right|dt$$

X12CrMoWVNbN10-1-1 steel has a body-centered cubic crystalline structure [31,32], where the main slip plane in a grain is {110}, and the slip direction is the diagonal of the cube <111>. There are 12 main slip systems and 36 secondary slip systems, hence the value of *n* in Equation (3) is 48.

### 2.2. Grain Boundary Cavitation Model

Experimental and theoretical studies [33,34] have shown that creep deformation and fracture are usually accompanied by the phenomenon of grain boundary cavitation at high temperatures. The damage is caused by creep cavitation at the grain boundary, including (1) the nucleation of cavities; (2) the growth of cavities; (3) the coalescence of adjacent cavities.

It is complicated to simulate the nucleation, growth, and coalescence of the cavities at the grain boundary during creep process. Therefore, in this study, for the purpose of convenience, the damage model adopts the implicit representation of grain boundary cavities, which can be studied by the method of continuum mechanics.

#### 2.2.1. Cavity Nucleation

**Figure 1:**
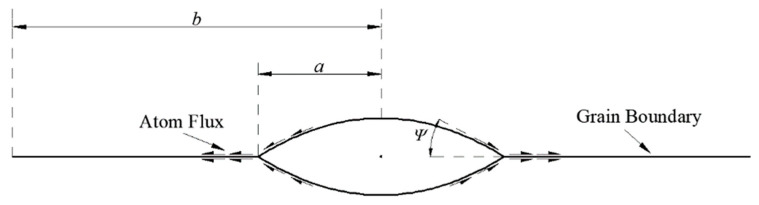
Idealized grain boundary cavity.

On the atomic scale, cavity nucleation occurs and is related to the local stress and grain boundary microstructure. Researchers [35,36] have investigated the mechanism of cavity nucleation at grain boundaries at high temperatures for decades. However, no unified theory can be used to portray the complex process of cavity nucleation. Therefore, this paper adopts the phenomenological model proposed by Tvergaard [37]. The schematic diagram of the model is shown in Figure 1. In accordance with this model, the cavity nucleation rate $\dot{\bar{N}}$, depending on the normal stress $\sigma_{n}$ at the grain boundary and the equivalent creep strain rate ${\dot{\varepsilon }}^{c}{}_{e}$, is governed by:

(11) $$\dot{\bar{N}}=F_{n}{\left(\frac{\sigma_{n}}{\sum_{0}}\right)}^{2}{\dot{\varepsilon }}^{c}{}_{e}\;\mathrm{for}\;\sigma_{n}>0$$

where $F_{n}$ is the nucleation rate parameter, $\sum_{0}$ is the traction normalization parameter and ${\dot{\varepsilon }}^{c}{}_{e}$ is the equivalent creep strain rate. However, the experimental data shows that the nucleation will not occur until sufficient inelastic deformation has been accumulated. To illustrate the phenomenon, Onck and Giessen [38] proposed a parameter related to accumulated creep strain and the normal stress:

(12) $$S=F_{n}{\left(\frac{\sigma_{n}}{\sum_{0}}\right)}^{2}\varepsilon_{e}^{c}\;\mathrm{for}\;\sigma_{n}>0$$

when S exceeds the threshold value $S_{thr}$, that is, when the stress level and creep strain in the grain boundary accumulate to a certain extent, it is considered that nucleation of cavities has occurred there. The threshold value $S_{thr}$ is inversely proportional to the initial cavity density ${\bar{N}}_{i}$, and the expression is written as follows:

(13) $$S_{thr}={\bar{N}}_{i}/F_{n}$$

where the initial cavity density ${\bar{N}}_{i}$ can be expressed by the initial distance $b_{0}$ between adjacent cavity:

(14) $${\bar{N}}_{i}=\frac{1}{\pi b_{0}^{2}}$$

In Equation (12), the accumulated creep strain $\varepsilon_{e}^{c}$ is expressed as:

(15) $$\varepsilon_{e}^{c}=\int_{0}^{t}{\dot{\varepsilon }}_{e}^{c}\;dt$$

This paper simplifies the model, that is, the new nucleated cavity has the same radius, *a*, as the current cavity does, and the half-distance, *b*, between adjacent cavities shown in Figure 1 can be expressed as:

(16) $$b=\frac{1}{\sqrt{\pi \bar{N}}}$$

where $\bar{N}$ is the cavity density.

#### 2.2.2. Cavity Growth

The creep deformation of the surrounding grains and the diffusion of atoms at the grain boundary depend on volume growth of cavity. The rate of volume growth $\dot{V}$ of the cavity at the grain boundary can be expressed as:

(17) $$\dot{V}={\dot{V}}_{d}+{\dot{V}}_{c}$$

where ${\dot{V}}_{d}$ and ${\dot{V}}_{c}$ respectively represent the contribution of atomic diffusion and grain creep at the grain boundary to the growth of the cavity volume, and the expressions [38,39] are shown as follows:

(18) $${\dot{V}}_{d}=4\pi D\frac{\sigma_{n}-(1-f)\sigma_{s}}{\ln\left(\frac{1}{f}\right)-\frac{1}{2}(3-f)(1-f)}$$

(19) $${\dot{V}}_{c}=\{\begin{array}{l} \pm 2\pi {\dot{\varepsilon }}_{e}^{c}a^{3}h(\psi ){\left[\alpha_{n}\left|\frac{\sigma_{m}}{\sigma_{e}}\right|+\beta_{n}\right]}^{m},\;\;\mathrm{for}\;\;\pm \frac{\sigma_{m}}{\sigma_{e}}>1\\ 2\pi {\dot{\varepsilon }}_{e}^{c}a^{3}h(\psi ){\left[\alpha_{n}+\beta_{n}\right]}^{m}\frac{\sigma_{m}}{\sigma_{e}},\;\;\mathrm{for}\;\left|\frac{\sigma_{m}}{\sigma_{e}}\right|<1 \end{array}$$

With

(20) $${\dot{\varepsilon }}_{e}^{c}=\sqrt{\frac{2}{3}D^{p}:D^{p}}$$

(21) $$\sigma_{e}=\sqrt{\frac{3}{2}\sigma^{\prime }:\sigma^{\prime }}$$

(22) $$\sigma_{m}=\frac{1}{3}\mathrm{tr}(\sigma )$$

(23) $$\sigma^{\prime }=\sigma -\frac{1}{3}\mathrm{tr}(\sigma )I$$

(24) $$f=\max\left\{{\left(\frac{a}{b}\right)}^{2},{\left(\frac{a}{a+1.5L}\right)}^{2}\right\}$$

(25) $$L={\left(D\frac{\sigma_{e}}{{\dot{\varepsilon }}_{e}^{c}}\right)}^{1/3}$$

(26) $$\alpha_{n}=\frac{3}{2m}$$

(27) $$\beta_{n}=\frac{\left(m-1)(m+0.4319\right)}{m^{2}}$$

where tr(σ) is the trace of σ, I is the second-order identity tensor and $\sigma_{n}$ is the average normal stress in the vicinity of the cavities. It is noted that the von Mises stress $\sigma_{e}$, average stress $\sigma_{m}$ and the effective creep strain rate ${\dot{\varepsilon }}_{e}^{c}$ are obtained from adjacent grains rather than cavities. We divide the grain boundary of finite thickness between adjacent grains into two halves (belonging to each of the adjacent grains) to obtain the magnitudes of stress and strain. *L* is the length scale factor related to stress and temperature introduced by Rice and Needleman [40], and *D* is the diffusion coefficient, which can be defined as:

(28) $$D=\frac{D_{b}\delta_{b}\Omega }{kT}\exp\left(-\frac{Q_{b}}{kN_{A}T}\right)$$

where Ω is the atomic volume, $D_{b}\delta_{b}$ is the frequency pre-exponential, *Q_b_* is the activation energy of the grain boundary diffusion, *T* is the absolute temperature, *k* is the Boltzmann constant, and *N_A_* is the Avogadro’s constant.

In Equation (18), $\sigma_{s}$ is the sintering stress, which is neglected in this work because its value is very small [41]. In Equation (24), the characteristic length *L* represents the competitive relationship of the two cavity growth mechanisms of creep and diffusion, which usually depends on temperature and stress. When the ratio of *L* to the cavity radius *a* has a relatively large value, the cavity growth is dominated by grain boundary diffusion. Conversely, when *L/a* is small, the cavity growth is dominated by dislocation creep. As the value of *L/a* decreases, the cavity volume growth rate increases significantly.

The cavity volume can usually be expressed by the radius *a* and the spherical cap shape parameter *h*:

(29) $$V=\frac{4}{3}\pi a^{3}h(\psi )$$

where the shape parameters of the spherical cap can be a function of the cavity tip angle ψ:

(30) $$h(\psi )=\frac{\left[{\left(1+\cos\psi \right)}^{-1}-\frac{1}{2}\cos\psi \right]}{\sin\psi }$$

According to the observation in the experiment of van der Giessen and Tvergaard [42], the typical value of ψ during the growth of the cavity is 75°, which is adopted in this model.

In summary, the rate of cavity radius can be expressed as:

(31) $$\dot{a}=\frac{{\dot{V}}_{c}+{\dot{V}}_{d}}{4\pi a^{2}h(\varphi )}$$

The above-mentioned grain boundary cavitation model is a classical one. Recently, Messner et al. [43] and Zhang et al. [44,45] employed it to model creep fracture of creep-resistant ferritic steels, showing its computing capability of predicting the effects of microstructure, stress and temperature on creep damage.

The cavities nucleate and grow under the action of external force. When the condition of cavities coalescence (*a/b* = 0.8) is satisfied [42], the cavities at the grain boundary stop growing, which means that the grain boundary is completely destroyed by the creep cavity, so we define the accumulated creep damage $D_{c}$ as:

(32) $$D_{c}=\frac{a}{0.8b}$$

### 2.3. Fatigue Crack Initiation Model

Fatigue indicator parameters (FIPs) can be used to reflect the mesoscale deformation mechanism of fatigue crack initiation as well as the fatigue damage evolutions, as a means of correlating the local microstructure with the most likely locations of fatigue crack initiation, and have been a dominant approach to modelling fatigue crack initiation at the grain scale recently [46,47]. The basic assumption is that irreversible slip generated in the slip system will lead to fatigue damage. The FIP used in this study is the accumulated plastic slip proposed by Manonukul and Dunne [48], reflecting the irreversible plastic deformation. Under cyclic loading, the cumulative plastic strain increases due to the reciprocating slip of dislocations, which can be obtained by the following formula:

(33) $$FIP_{p}=\int_{0}^{t}{\left(\frac{2}{3}D^{p}:D^{p}\right)}^{\frac{1}{2}}dt$$

In this study, it is defined that the critical value of the accumulated equivalent plastic strain *FIP_p_* is *FIP_p,crit_*, i.e., when *FIP_p_* = *FIP_p,crit_*, the fatigue crack initiates. Therefore, based on this concept, the accumulated fatigue damage $D_{f}$ can be defined as:

(34) $$D_{f}=\frac{FIP_{p}}{FIP_{p}{}_{,crit}}$$

## 3. Finite Element Implementation

### 3.1. Validation of Model Parameters

The single crystal plasticity model mentioned in the previous section contains 14 material parameters. The crystal plasticity parameters of X12CrMoWVNbN10-1-1 at 600 °C are obtained according to the data reported by Zhao et al. [31] and Li et al. [49]. Table 1 gives the parameters used in the crystal plasticity constitutive model.

The damage model parameters of grain boundary cavity coalescence are mainly obtained by Wen et al. [13,41] and Messner et al. [43]. All the parameters are listed in Table 2. In order to calibrate the parameters of the model and verify the feasibility of the proposed model, a polycrystalline RVE model of 400 μm × 400 μm that can distinguish grain boundaries and grains is established. It consists of 100 grains with random grain orientations, with CPE4 elements (a four-node bilinear plane strain quadrilateral element) used in the mesh. Periodic boundary conditions are used to simulate the creep process at 301.4 MPa and 220 MPa. In order to obtain macro-mechanical properties that are easy to compare, the strain is homogenized:

(35) $$\bar{\varepsilon }=\frac{1}{V}\int_{V}\varepsilon dV$$

**Figure 2:**
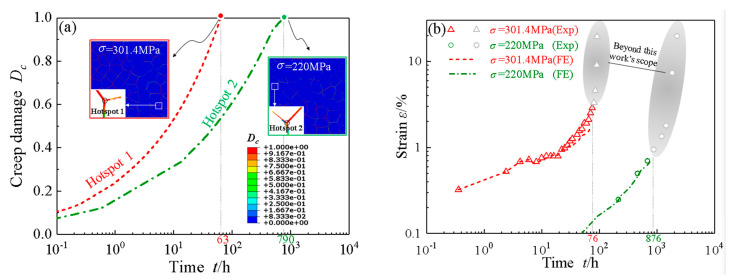
(**a**) Evolution curves of creep damage vs. time of hotspot 1 and 2 and the corresponding contour plots of creep damage; (**b**) Comparison of creep strain vs. time curves between experimental results [31] and simulations at stress of 301.4 MPa and 220 MPa at 600 °C.

According to Ref [40], it is considered that the coalescence of cavities first occurs due to the break of the ligaments between the adjacent cavities, where creep damage equals to *a*/0.8*b* in Equation (32). The hotspot for crack initiation is defined as the creep or fatigue damage firstly accumulating to 1 in the model in the numerical aspects, the physical meaning behind which is the initial coalescence of cavities for the investigated material. Both hotspot 1 at 301.4 MPa and hotspot 2 at 220 MPa are located at the triple grain boundary junctions, as respectively seen in the contours in Figure 2a. The full-length creep damage evolution curves regarding hotspot 1 and 2 are backtracked and plotted in Figure 2a as well, which increase with the increase of creep time. The simulated creep crack initiation times ($D_{c}$ = 1) are detected as 63 h for hotspot 1 and 790 h for hotspot 2. The simulated creep crack initiation times approach but are relatively shorter than the experimental transition times, 76 h and 876 h at 301.4 MPa and 220 MPa, respectively, in Figure 2b. The small difference in values lies in that the simulated creep crack initiation is defined as the first cavity coalescence in the hotspots, while a certain number of cavity coalescences are required at the experimental transition time. Therefore, both the creep crack initiation sites and creep crack initiation times are well predicted at two typical stress levels in our simulations.

### 3.2. Simulation of Crack Initiation of Notched Specimens under Creep-Fatigue Loading

**Figure 3:**
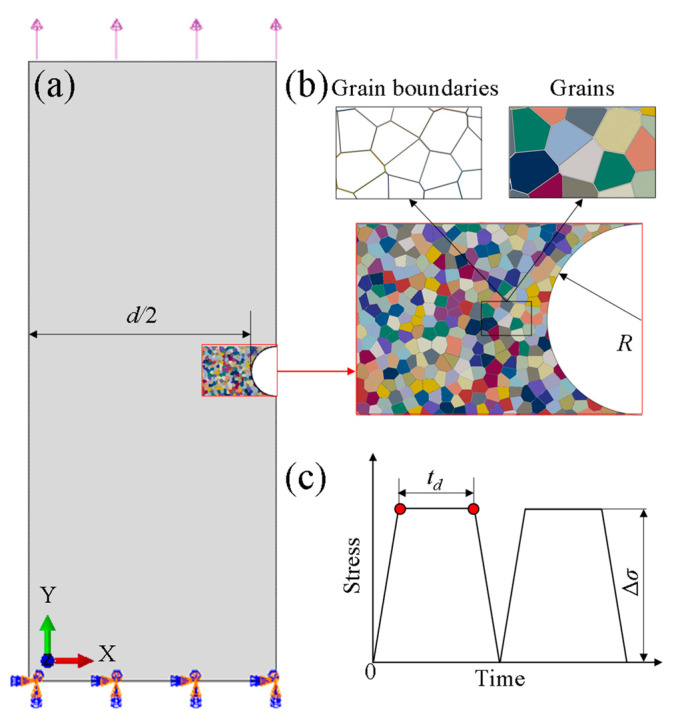
(**a**) Overall finite element model of notch specimen, (**b**) sub-model for crystal plasticity around the notch area and (**c**) creep-fatigue loading waveform.

In this paper, notched specimens are used to simulate crack initiation. The geometry and dimensions are shown in Figure 3a. The overall size of the model is 2000 μm × 5000 μm, and the notch area is 400 μm × 600 μm. A multiscale numerical framework combining crystal plasticity model and damage model of cavity coalescence at grain boundaries is proposed. As creep-fatigue damage is likely to accumulate in the notch area, in order to save calculation cost, the sub-model for crystal plasticity is established only in the notch area, see Figure 3a. It should be noted that X12CrMoWVNbN10-1-1 steel is a polycrystalline metal with a complex microstructure and may involve multiple phases such as ferrite, austenite, martensite, and carbide. However, the paper is focused on the effects of grains and grain boundaries on the creep-fatigue crack initiation of polycrystalline metallic materials. The complex microstructure is not fully considered in the work. Based on the Voronoi method, an idealized microstructure containing grains of a certain average grain size (AGS) and finite thickness of grain boundaries is generated in the notch area as shown in Figure 3b. In the overall finite element model of the specimen with the initial notch, the boundary conditions are no longer characterized by periodicity. Instead, Boolean operations are performed on the sub-model and the macroscale model. A total of about 25,000–50,000 plane strain elements are generated and more than 90% of the elements are concentrated in Figure 3b. The transition mesh is used in the transition area of grains and corresponding grain boundaries. In this model, a mesoscopic sub-model is established to distinguish the grain boundaries, and different material properties are assigned to the grains and the grain boundaries, respectively. At the grain boundaries, the creep damage model of the coalescence of cavities is used to predict the creep crack initiation in the sub-model. Meanwhile, the fatigue damage mentioned in the previous section is used to reflect the fatigue crack initiation in the sub-model. Boundary conditions and load waveform are shown in Figure 3c. The boundary condition of clamped support is set for the lower surface, and the creep-fatigue load under stress ratio of 0 with holding time of *t_d_* is set for the upper surface.

## 4. Results and Discussion

### 4.1. Effect of Initial Notch Size on Creep and Fatigue Crack Initiations

To investigate the influence of the initial notch size on creep and fatigue crack initiations, the microstructures of three different initial notch sizes are modeled in the section. The notches are uniformly set as semicircular notches with the initial radii of 50 μm, 100 μm and 200 μm and the notch acuity ratios (ratio of the plate’s net section width to the notch root radius, *d*/*R*) of 78, 38, and 18 in sequence, and the AGS is set to 50 μm. Stress range of cyclic load is 300 MPa and holding time *t_d_* = 300 s.

In addition, for a given initial notch size, two types of sub-models with different grain orientations are built, in order to exclude the effects of microstructures and grain orientations. The above-mentioned models have the same microstructure morphology but are assigned different random orientation distributions to the grains. To facilitate the description, the models with notch root radii of 200 μm, 100 μm, and 50 μm are numbered as A, B, and C in order, and the different random orientation distributions are divided into 2 groups, noted as subscript 1 and 2. The fatigue damage *D_f_* and the creep damage *D_c_* of grain boundary cavity coalescence at grain boundaries are then used to predict the initiations of fatigue and creep crack, respectively.

**Figure 4:**
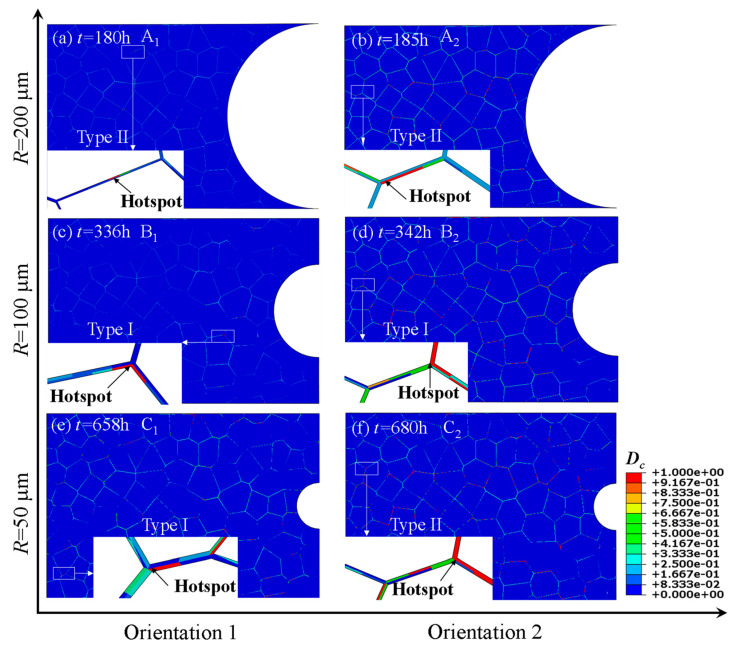
Contour plots of creep damage *D_c_*: (**a**) Model A_1_ (*R* = 200 μm, Orientation 1); (**b**) Model A_2_ (*R* = 200 μm, Orientation 2); (**c**) Model B_1_(*R* = 100 μm, Orientation 1); (**d**) Model B_2_ (*R* = 100 μm, Orientation 2); (**e**) Model C_1_ (*R* = 50 μm, Orientation 1); (**f**) Model C_2_ (*R* = 50 μm, Orientation 2).

**Figure 5:**
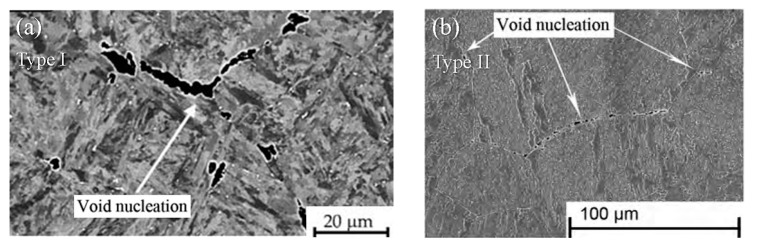
Nucleation and coalescence of cavities under creep conditions at (**a**) the triple junction (Type I, e.g., 9Cr1Mo-NbV [50]), and (**b**) the grain boundaries perpendicular (or almost perpendicular) to the loading direction (Type II, e.g., 25Cr2NiMo1V [51]).

As seen from the contour plots of creep damage in Figure 4, the coalescence of cavities mostly occurs at the triple grain boundary junction (Type I) and grain boundaries that are approximately perpendicular to the loading direction (Type II). In order to explore the reasons for this phenomenon, some typical grain boundaries are enlarged for further investigations. It can be found that the maximum principal stress of the grain boundary approximately perpendicular to the loading direction is much higher than that of the grain boundary approximately parallel to the loading direction. Since the cavity volume growth rate controlled by diffusion is mainly affected by the maximum principal stress at the grain boundary, the volume of the cavities at the grain boundary that is approximately perpendicular to the loading direction grows faster, and the cavity coalescence is prone to occur. The stress concentration is more prone to occur at the triple grain boundary junction and the diffusion mechanism controlled by the principal stress has a great impact on the growth of cavities at the grain boundary approximately perpendicular to the loading direction, both of which accelerate coalescence of cavities. The modelling can be supported by experimental observations for Cr-Mo-V steels, 9Cr1Mo-NbV and 25Cr2NiMo1V. As shown in Figure 5, the nucleation and coalescence of cavities occurred at the triple junction (Type I) [50] or at the grain boundaries approximately perpendicular to the loading direction (Type II) [51].

**Figure 6:**
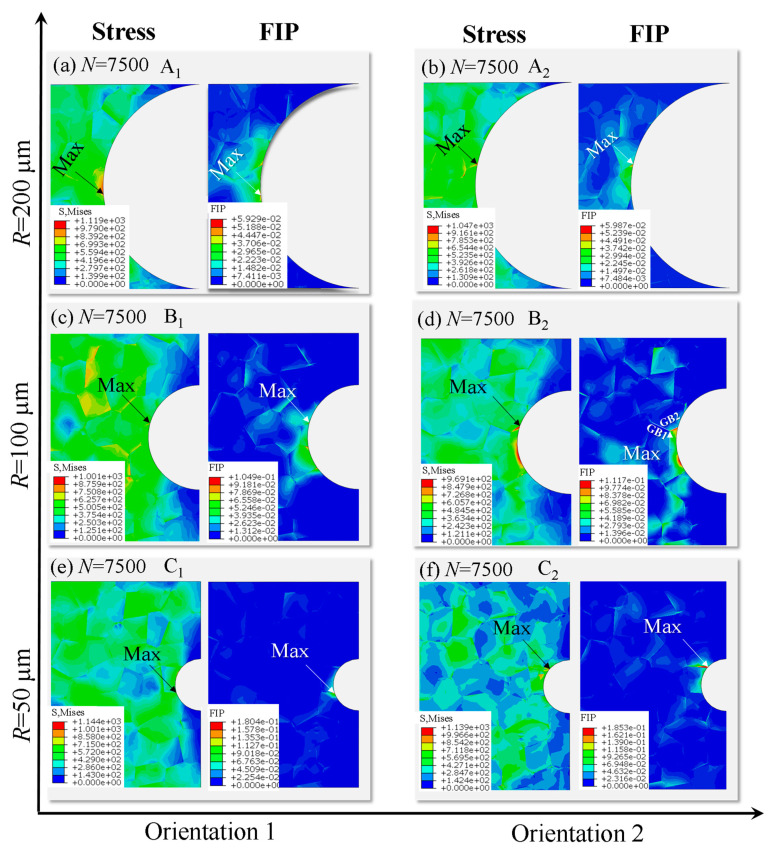
Distributions of von Mises stress and acumulative equivalent plastic strain *FIP_p_* (**a**) Model A_1_ (*R* = 200 μm, Orientation 1); (**b**) Model A_2_ (*R* = 200 μm, Orientation 2); (**c**) Model B_1_(*R* = 100 μm, Orientation 1); (**d**) Model B_2_ (*R* = 100 μm, Orientation 2); (**e**) Model C_1_(*R* = 50 μm, Orientation 1); (**f**) Model C_2_ (*R* = 50 μm, Orientation 2).

In the aspect of fatigue damage, the predicted results based on equivalent cumulative plastic strain *FIP_p_* and von Mises stress for specimens with different initial notch sizes are exhibited in Figure 6. It can be seen that although the positions of crack initiation in the models of different orientations are not exactly the same, they are basically located at the grain boundaries on the notch surface of the specimen.

By comparing the corresponding von Mises stress distributions from Figure 6, it is found that the location of the fatigue crack initiation is almost the same as that of the maximum stress, mainly because high stress produces local yield on the surface of the notch, resulting in higher accumulated fatigue damage. The crack initiation positions of A_1_ and A_2_ specimens appear at the two grain boundaries of the same grain at the notch and those of C_1_ and C_2_ specimens are also in relatively the same situation, approximately perpendicular to the tensile direction. Differently, although the fatigue crack initiation of B_1_ and B_2_ appears at the same grain boundary on the notch surface, the positions are respectively the grain boundary on the notch surface and the triple grain boundary junctions.

Through the analysis of grain orientation at B_1_ and B_2_, it is found that there is a significant orientation difference between the grain boundaries GB1 and GB2 in B_2_. From the stress distribution in Figure 6b, it can be seen that the stress of the grain boundary GB1 is very small, and the stress concentration phenomenon of the grain boundary GB2 at the position of the notch surface is very obvious. Since the orientation is relatively easy to slip, the crack initiates at the junction of the grain boundary GB1 and GB2. It can be observed from the value of the accumulated equivalent plastic strain that the difference of specimens with the same notch size is small although different random orientations are given.

**Figure 7:**
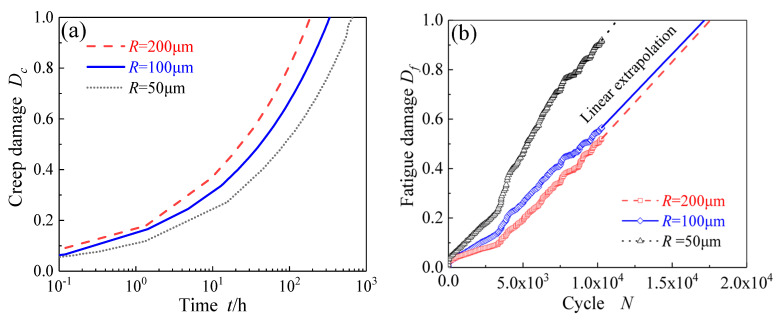
Evolution curves of (**a**) creep damage vs. time, and (**b**) fatigue damage vs. cycle of the hotspots in the specimens with different notch root radii.

Figure 7a shows the evolution curves of creep damage of hotspot in specimens with different notch root radii, which exhibits that the larger the notch acuity ratio is, the later the creep crack initiates. A similar phenomenon was found in creep experiments on notched specimens of CrMo steel [52,53], where the failure time was prolonged with increasing notch acuity ratio due to the enhanced notch strengthening effect of CrMo steel.

The elongation of X12CrMoWVNbN10-1-1 is 23% based on the tensile experiment at 600 °C [49]. Due to the limitation of calculation efficiency and the assumption of linear accumulation of equivalent plastic strain, the evolution curve of *D_f_* for cycle is linearly extended to *D_f_* = 1, as shown in Figure 7b, which is consistent with the tendency in many published literature [54,55]. From the evolution curves of fatigue damage of the hotspots in the specimens with different notch root radii, it can be found that the number, *N*, of cycles to fatigue crack initiation is reduced as the radius of the root of the notch decreases.

### 4.2. Effect of Stress Range on Creep and Fatigue Crack Initiations

**Figure 8:**
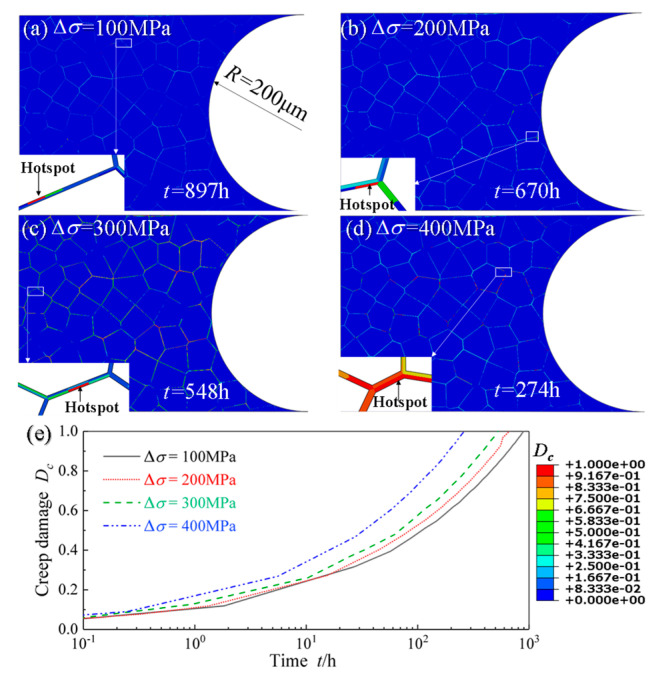
Distribution of creep damage *D_c_* of notch specimen (*R* = 200 μm) with 4 stress ranges: (**a**) 100 MPa; (**b**) 200 MPa; (**c**) 300 MPa; (**d**) 400 MPa and (**e**) the evolution curves of *D_c_* of the hotsopts from (**a**–**d**).

Figure 8a–d show the contour plots of creep damage under different stress ranges, exhibiting that the coalescence of cavities mostly occurs at the triple grain boundary junctions and the grain boundaries approximately perpendicular to the loading direction. The evolution curves of creep damage of hotspots with time under different stress ranges are shown in Figure 8e, from which it can be obtained that the time of creep crack initiation is reduced as the stress range increases.

**Figure 9:**
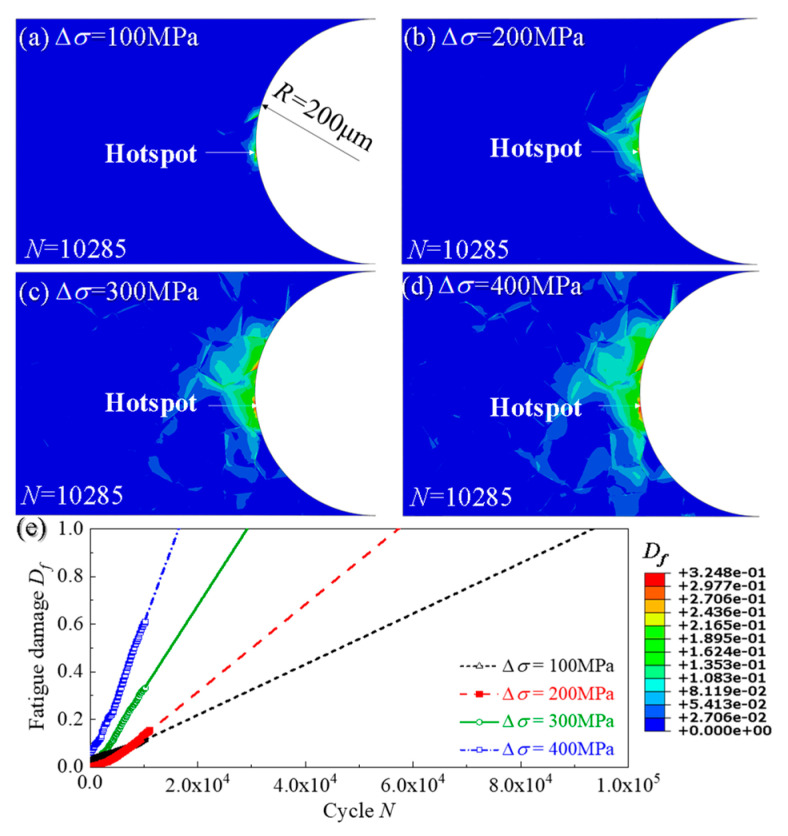
Distribution of fatigue damage of notch specimen (*R* = 200 μm) with 4 stress ranges: (**a**) 100 MPa; (**b**) 200 MPa; (**c**) 300 MPa; (**d**) 400 MPa and (**e**) the evolution curves of *D_f_* of the hotsopts from (**a**–**d**).

Figure 9a–d show the distributions of fatigue damage in the sub-models undergoing 10,285-cycle load at different stress ranges. Similarly, Figure 9e presents the evolution curves of fatigue damage of the selected hotspots. It can be seen that, despite the different stress ranges, the hotspots with the highest value of fatigue damage (i.e., the location of fatigue crack initiation) are always located at the grain boundary on the notch surface. However, as the stress range increases, the value of fatigue damage increases in the same load cycle, meaning that the increase of the stress range shortens the fatigue crack initiation life.

### 4.3. Effect of Average Grain Size on Creep and Fatigue Crack Initiations

In this section, the effect of AGS on cavity nucleation and creep-fatigue crack initiation is investigated by modeling four different AGSs of notches.

**Figure 10:**
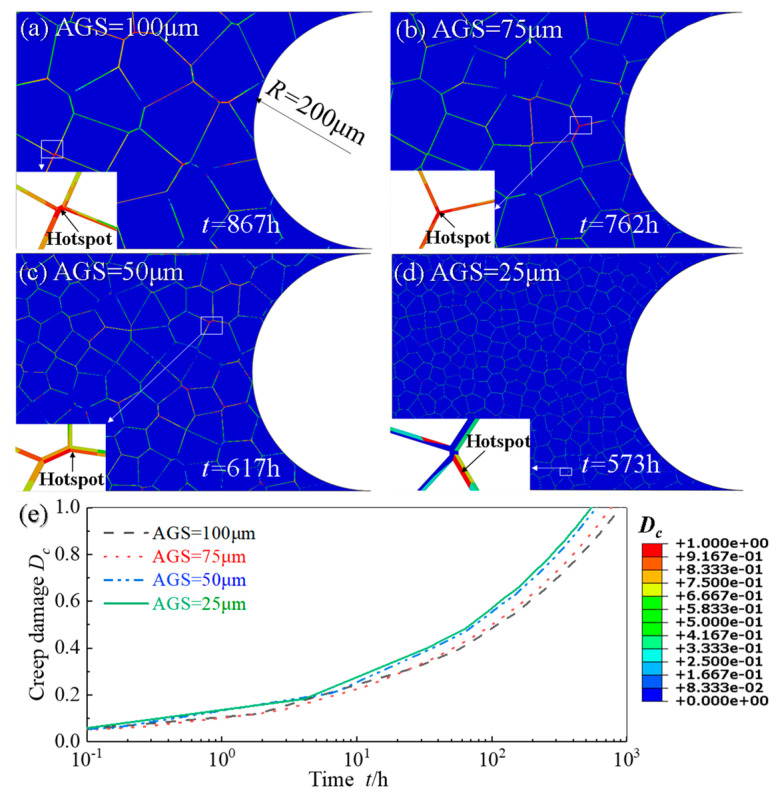
Distribution of creep damage of notch specimens with different AGSs: (**a**) 100 μm; (**b**) 75 μm; (**c**) 50 μm; (**d**) 25 μm and (**e**) the evolution curves of *D_c_* of hotsopts from (**a**–**d**).

Figure 10a–d show the contour plots of creep damage *D_c_* in the sub-models with four different AGSs, exhibiting that the coalescence of cavities mostly occurs at the triple grain boundary junction and the grain boundaries perpendicular to the loading direction. The evolution curves of creep damage of hotspots of the specimens with four different AGSs are shown in Figure 10e, from which it can be obtained that the time of creep crack initiation is shortened as the AGS decreases from 100 μm to 25 μm. This is due to the fact that as the grain size decreases, the proportion of grain boundaries increases significantly for the same sub-model. Since creep damage is mainly caused by the growth of cavities on grain boundaries, an increase in the fraction of grain boundaries will lead to a greater creep damage rate, which is also demonstrated in the studies [56,57].

**Figure 11:**
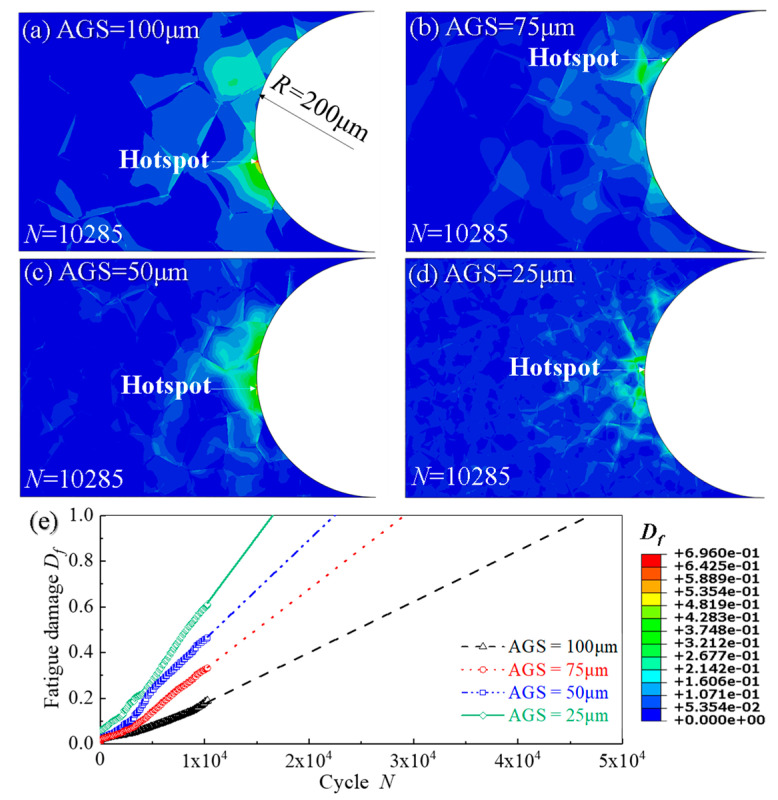
Distribution of fatigue damage of notch specimens with different AGSs: (**a**) 25 μm; (**b**) 50 μm; (**c**) 75 μm; (**d**) 100 μm and (**e**) the evolution curves of fatigue damage *D_f_* of hotspots from (**a**–**d**).

Figure 11a–d show the contour plots of fatigue damage *D_f_* in the sub-models with four different AGSs when *N* = 10,285, exhibiting that fatigue crack initiation locates at the grain boundary on the notch surface. The evolution curves of creep damage of hotspots of the specimens with four different AGSs are shown in Figure 10e, from which it can be obtained that fatigue damage decreases, indicating that the life of fatigue crack initiation is extended as the AGS decreases from 100 μm to 25 μm. Consistent with the phenomenon, the increase of the AGS prolongs the time of fatigue crack initiation, observed in the experiments of Qin et al. [58].

### 4.4. Effect of Holding Time on Creep and Fatigue Crack Initiations

**Figure 12:**
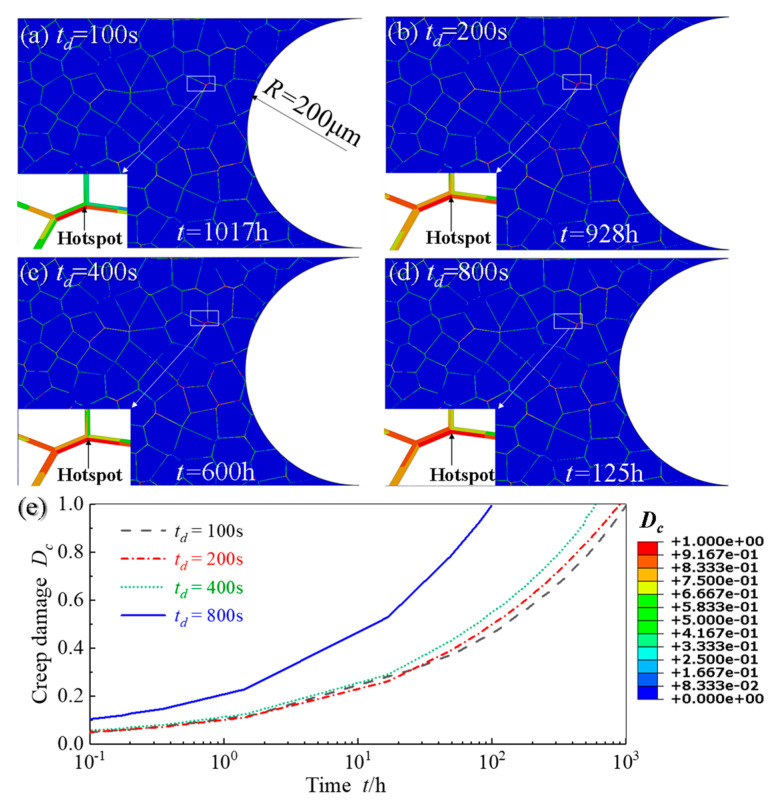
Distribution of creep damage distribution of notch specimens for four different holding time: (**a**) *t_d_* = 100 s; (**b**) *t_d_* = 200 s; (**c**) *t_d_* = 400 s; (**d**) *t_d_* = 800 s and (**e**) the evolution curves of creep damage *D_c_* of hotsopts from (**a**–**d**).

**Figure 13:**
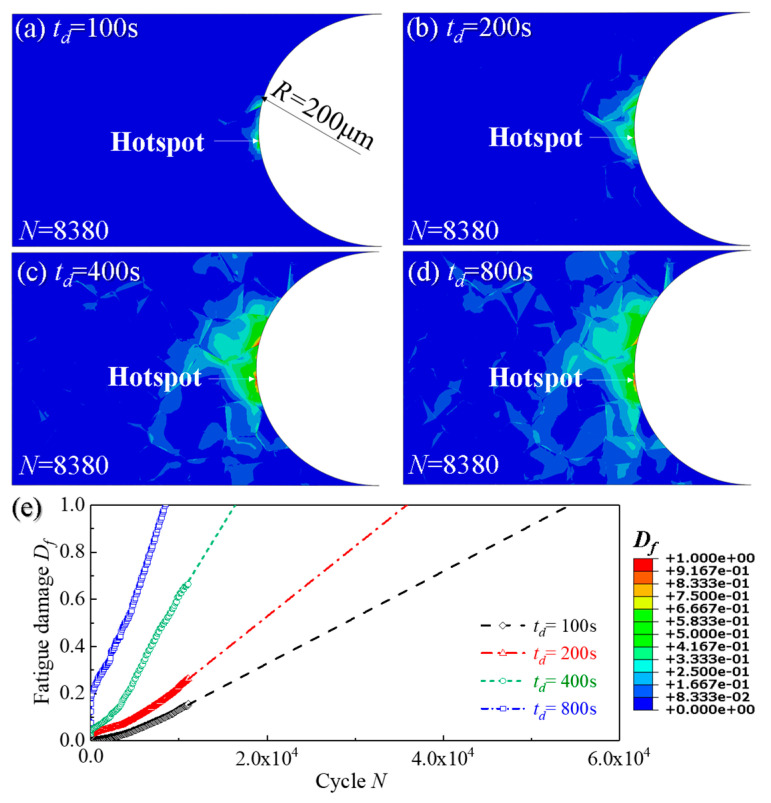
Distribution of fatigue damage of notch specimens for four different holding time: (**a**) *t_d_* = 100 s; (**b**) *t_d_* = 200 s; (**c**) *t_d_* = 400 s; (**d**) *t_d_* = 800 s and (**e**) the evolution curves of fatigue damage *D_f_* of hotspots from (**a**–**d**).

In this section, in order to explore the effect of holding time on creep and fatigue crack initiations, the sub-model with a notch root radius of 200 μm and AGS of 50 μm is simulated. The stress range is 200 MPa and four holding times are 100 s, 200 s, 400 s and 800 s, respectively. Figure 12 and Figure 13 respectively show the damage distribution and locations of creep and fatigue crack initiation under cyclic loadings with four different holding times. It can be found that the locations of creep crack initiation are independent on the holding time, as shown in Figure 12a–d. Similar results are also observed for fatigue crack initiation locations from Figure 13a–d. The difference between Figure 12 and Figure 13 lies in that the creep cracks initiate at the triple grain boundary junction at a distance of about two grains from the surface of the notch, while the fatigue cracks initiate at the triple grain boundary junction on the surface of the notch.

Figure 12e shows the evolution of creep damage *D_c_* of hotspot with time under four different holding times, where the creep crack initiation times are different. By comparing the creep crack initiation time under four different holding times, it can be found that with the increase of holding time, the corresponding creep damage is also gradually increased. In particular, creep crack initiation time is significantly advanced when the holding time is long enough.

It can be found from Figure 13e that fatigue cracks initiate after experiencing fewer cycles as the holding time increases. The results are also in relatively agreement with the findings [59,60,61], where the introduction of longer holding times at the peak stress resulted in higher magnitudes of plastic slip rate, the subsequent equivalent plastic strain and the final *FIP_p_*.

**Figure 14:**
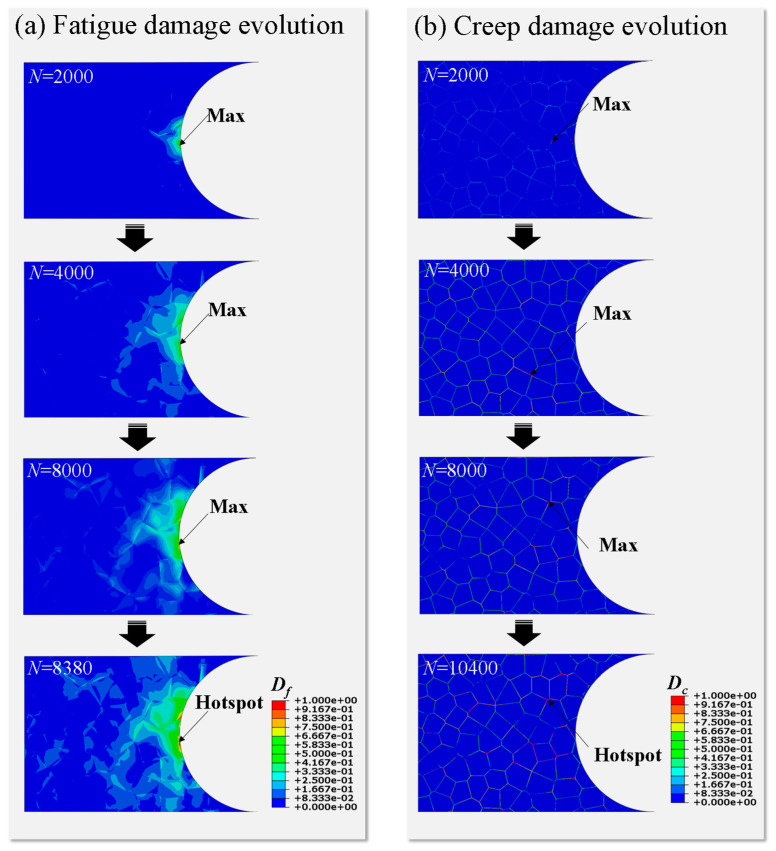
Evolution of (**a**) fatigue and (**b**) creep damage for holding time *t_d_* = 800 s.

To illustrate the evolutions of fatigue damage and creep damage at grain boundaries in the sub-model, the holding time of 800 s is used as an example, as respectively shown in Figure 14a,b. It can be seen from Figure 14a that the accumulated equivalent plastic strain and fatigue damage are concentrated at the same location of notched surface during the whole fatigue lifetime. When *N* = 8380, the maximum of fatigue damage reaches a critical value, indicating that fatigue crack initiation occurs on the notched surface. It can be seen from Figure 14b that the locations of maximum creep damage always transfer before *N* = 8000. In addition, creep damage in the sub-model around the notch begins to appear at a limited number of grain boundaries when it undergoes 2000 cycles of loading. As the number of cycles increases, the overall damage accumulates at the grain boundaries to greater extent. In particular, the accumulations of damage at the triple grain boundary junctions and at grain boundaries approximately perpendicular to the loading direction are more significant. With the number of cycles increasing to 10,400, maximum creep damage at hotspot accumulates to 1 and the creep crack initiates.

## 5. Conclusions

In this paper, a multiscale numerical framework covering crystal plasticity and grain boundary cavitation is proposed. Predictions of the initiations of creep and fatigue cracks in notched specimens of X12CrMoWVNbN10-1-1 steel are achieved by the numerical methods. The creep crack initiation is predicted by the cavity coalescence damage model at grain boundary, while the fatigue crack initiation is predicted by accumulated equivalent plastic strain both at grain and grain boundary. The effects of stress range, AGS and holding time on creep and fatigue crack initiations are investigated, and the following main conclusions are drawn:

1. Fatigue crack initiation occurs on the surface of the notch root during the whole lifetime. The creep crack initiation is basically located at a distance of 2~8 grains from the surface of the notch, at the triple grain boundary junction or at the grain boundary approximately perpendicular to the loading direction;
2. For specimens with a notch root radius from 50 μm to 200 μm, the fatigue initiation time is shortened with the decrease of the root radius of the notch, while the creep crack initiation time becomes longer with the increase of the notch acuity ratio due to the reinforcement effect of the notch;
3. As the stress range increases, the initiation times of creep and fatigue crack initiations are shortened. Meanwhile, with the decrease of AGS and the increase of holding time, the initiations of creep and fatigue cracks also accelerate;
4. For specimens with the same notch size and stress range, both creep and fatigue crack initiation sites are insensitive to the holding times ranging from 100 s to 800 s.

## Figures and Tables

**Table 1:** Parameters of X12CrMoWVNbN10-1-1 related to crystal plasticity [31,49].

Category	Parameter	Unit	Value
Elastic modulus	*C*_11_	GPa	167.2
	*C*_12_	GPa	140.5
	*C*_44_	GPa	74.9
Material parameters in flow rule	Reference strain rate, ${\dot{\gamma }}_{0}$	s^−1^	0.001
	Flow rule power law exponent, *m*	/	30
Material parameters in self- and latent-isotropic hardening for the main slip systems	Initial hardening modulus, $h_{0}$	MPa	1340
	Initial slip system strength, $\tau_{0}$	MPa	150
	Critical shear stress, $\tau_{s}$	MPa	270
Material parameters in self- and latent-isotropic hardening for the secondary slip systems	Initial hardening modulus, $h_{0}$	MPa	6700
	Initial slip system strength, $\tau_{0}$	MPa	1350
	Critical shear stress, $\tau_{s}$	MPa	750
Material parameters in kinematic hardening	Direct hardening coefficient, *c*	MPa	5800
	Dynamic response coefficient, *d*	/	2
Critical value of *FIP_p_*	*FIP_p,crit_*	/	0.23

**Table 2:** Parameters of X12CrMoWVNbN10-1-1 related to cavity coalescence damage model [13,41,43].

Parameter	Unit	Value
Initial cavity radius, $a_{0}$	mm	5 × 10^−5^
Half initial distance between cavities, $b_{0}$	mm	6 × 10^−2^
Atomic volume, Ω	m^3^	1.18 × 10^−29^
Grain boundary diffusion parameter, D	mm^5^/N/min	4.75 × 10^−15^
Activation energy for grain boundary diffusion, $Q_{b}$	kJ/mol	174
pre-exponent diffusion coefficient, $D_{b}\delta_{b}$	mm^3^/s	1.35 × 10^−20^
Traction normalization parameter, $\sum_{0}$	MPa	100
Equilibrium cavity tip half-angle, ψ	°	75
Nucleation rate constant, $F_{n}$	mm^−2^	5.6 × 10^−16^

## Data Availability

The raw/processed data required to reproduce these findings cannot be shared at this time as the data also forms part of an ongoing study.
